# Evaluating the In-vitro Antibacterial Effect of Iranian Propolis on Oral Microorganisms

**Published:** 2011

**Authors:** Tahereh Sadat Jafarzadeh Kashi, Ruha Kasra Kermanshahi, Mohammad Erfan, Elahe Vahid Dastjerdi, Yashar Rezaei, Fahimeh Sadat Tabatabaei

**Affiliations:** a *Department of Dental Materials,Faculty of Dentistry, Tehran University of Medical Sciences, Tehran, Iran. *; b *Research Center for Medical and Technology in Medical Sciences, Tehran University of Medical Sciences, Tehran, Iran*; c *Departement of Biology, Azzahra University, Tehran, Iran.*; d *Department of Pharmaceutics, Pharmacy School, ShahidBeheshtiUniversityofMedicalSciences, Tehran, Iran *; e *Department of Orthodontics,Dental School, ShahidBeheshti University of Medical Sciences, Tehran, Iran. *; f *Department of Dental Materials,Dental School, Shahid Beheshti University of Medical Sciences, Tehran, Iran.*

**Keywords:** Iranian propolis, Ethanolic extracts of propolis, Water extract of propolis, Oral microorganisms

## Abstract

Propolis has traditionally been used in curing infections and healing wounds and burns. Current researches have shown that propolis has antibacterial, antifungal and antiviral actions however, the pharmacological activity of propolis is highly variable depending on its geographic origin. There have been few studies on the effects of Iranian propolis on the oral microorganisms. In this *in-vitro* study, the antimicrobial activity of the ethanolic and water extracts of the Iranian propolis (10%, w/v) from north-east area of Tehran was evaluated. Susceptibility of the oral strains tested (*Streptococcus mutans* ATCC 35668; *Streptococcus salivarius* ATCC 9222; *Staphylococcus aureus* ATCC 25923; *Enterococcus faecalis* ATCC 9854 and *Lactobacillus casei *ATCC 39392) was evaluated using the agar diffusion method at a concentration of 20 mg/mL of propolis and the zones of growth inhibition were measured. Antibacterial activity was determined by using minimum inhibitory concentration (MIC) and minimum bactericidal concentration (MBC) at different concentrations of propolis. The ethanolic extract showed bacteriostatic and bactericidal activity against all the strains, with MIC and MBC ranges of 250-500 µg/mL. The MIC concentration of the water extract was 500 µg/mL against *S. mutans* and *E. faecalis*. The water extract showed bactericidal activity only against *S. mutans* (20 mg/mL). These results indicate that the ethanolic extract is probably more useful in the control of oral biofilms and subsequent dental caries development. However, to determine the consequence of the ethanolic extract of Iranian propolis on the oral mucosa, *in-vivo* studies of its possible effects are needed.

## Introduction

Propolis is a natural non-toxic beehive product, which is used for building and restoration of the honeycomb (1). The term propolis comes from the Greek ‘pro’, in front, ‘polis’ means ‘town’ or ‘city’ and bees use propolis to seal their hives against the attack of the other insects (2). In the hive, propolis act as a biocide, being active against the invasive bacteria, fungi and even invading larvae (3). Other biological activities have also been depicted for propolis, including antibacterial (4), antifungal (5), antiviral (6), antitumor (7), immunomodulation (8) and anti-inflammatory (9) activities. The antimicrobial activity of propolis ethanolic extract of different geographic origin against oral pathogens has been studied by several authors (4, 10-12); however, few studies have investigated on Iranian propolis (13-16).

Dental plaque, an oral biofilm that is formed on the tooth surface (17), plays an important role in the etiology of dental caries and periodontal disease (18). Thus, the control of dental plaque is one of the targets for the prevention of dental caries and periodontal disease. Several different approaches have been proposed for controlling the biofilm, (19) such as: prevention of biofilm formation, disruption of existing biofilms, avoidance of further biofilm growth, and assassination of microorganisms in the biofilm. Among these approaches, the use of anti-biofilm agents that not only reduce the viability, but also control the colonization and accumulation of cariogenic bacteria on the tooth surface could be more effective.

Chlorhexidine is an anti-biofilm agent that is clinically efficient on a wide range of microorganisms in the oral cavity (20). However, the use of chlorhexidine, as an anti-caries agent, not only remains controversial (21) but also has common side effects, including the formation of extrinsic stain on the tooth and tongue (22). As a result, there is a huge interest in the development of new anti-biofilm agents. Several plants and natural product have been studied for their potential in the prevention of dental caries (21, 23). Despite increasing the use of propolis worldwide, only a few studies have been carried out to determine the inhibitory effect of Iranian propolis against some bacteria of relevancein dentistry (24, 25). Within this context, the aim of this study was to evaluate the antibacterial activity of the ethanolic and water extracts of Iranian propolis against several oral pathogens. 

## Experimental

*Extraction of propolis*

Propolis sample was collected from colonies of honeybees located in the north-east area of Tehran in Iran. Hand collected propolis was kept in a dry place and stored at 4^°^C until its complete process. The sample was chopped into small pieces, ground to a fine powder using a Moulinex blender and extracted with 80% ethanol (1 : 10 w/v) in a shaker at room temperature for 48 h. The ethanolic extract solution (EEP) was then filtered with Whatman No. 4 filter paper and concentrated in a rotary evaporator (Heidolph, Germany) to obtain the crude extract in paste form and kept in a dry and dark place.

For obtaining water extract of propolis (WEP), the dried and finely ground propolis was extracted with distilled water (1 : 10 w/v) by means of continuous stirring at room temperature for 48 h. After filtration, the solution was concentrated in a rotary evaporator to afford crude extract.

Propolis crude extracts were redissolved in 80% ethanol or distilled water at a concentration of 20 mg/mL. The extracts were then filtered and used for antibacterial testing.

*Bacterial strains*

The bacterial strains used in this study were *Streptococcus mutans *ATCC 35668, *Streptococcus salivarius* ATCC 9222, *Staphylococcus aureus* ATCC 25923, *Enterococcus faecalis* ATCC 9854 and *Lactobacillus casei *ATCC 39392. All microorganisms were provided in lyophilized form by Biotechnology Institute (Iranian Research Organization for Sciences and Technology, Tehran). All of these strains were used to determine the minimum inhibitory concentration (MIC) and the minimum bactericidal concentration (MBC). 

*Preparation of inoculums*

All bacteria were transferred from the stock cultures to tryptic soy agar (TSA) (Liofilchem, Teramo, Italy), blood agar (for* Streptococcus*) (Liofilchem, Teramo, Italy) and de Man, Rogosa and Sharpe (MRS) agar plates (for *L. casei*, Liofilchem, Teramo, Italy) and incubated overnight at 37°C. Single colonies from plates were transferred into TSB (Liofilchem, Teramo, Italy) and MRS broth (for *L. casei*, Liofilchem) and incubated at 37^°^C, for 24 or 48 h, for *L. casei* and used as inoculums. The turbidity of the suspension was adjusted spectrophotometrically to the McFarland 0.5 turbidity standard (1.5 × 10^8^ CFU/mL).

*Antimicrobial activity*

Antimicrobial activity of Iranian propolis extracts was investigated using agar diffusion method. Test plates (diameter 10 cm) were prepared with 20 mL of Mueller-Hinton agar (MHA) (Liofilchem, Teramo, Italy), and six wells of 8 mm in diameter were punched in the agar plates by using sterile glass-made pipettes attached to a vacuum pump. Sterile swabs were dipped into the bacterial suspension containing 1.5 × 10^8^ CFU/mL and inoculated on to plate surfaces. Each well was filled with 300 µL of the extracts or negative controls (80% ethanol and distilled water). Two wells without the extracts served as the positive control. The plates were kept for 2 h at room temperature to allow the diffusion of the agents through the agar. Afterwards, the plates were incubated at 37°C in an appropriate gaseous condition and for an appropriate period of time (aerobes, 24 h and *L.Casei*, 48 h in an anaerobic jar). Zones of inhibition of microbial growth around the holes were measured and recorded after the incubation time. The inhibitory zone was considered the shortest distance (mm) from the outside margin of the samples to the initial point of the microbial growth. All measurements were performed twice by the same blinded operator. Five replicates were made for each microorganism.

*Effects on viability of suspension cells*

The MIC was determined based on the macro-dilution tube methods (TSB or MRS broth for *L. Casei*) according to NCCLS M27-P (1990). For the determination of MIC, inoculum suspensions were prepared from 24 h broth cultures. Diluted 20 µL suspensions of each bacterial strain were added to 500 µL of various concentrations of propolis diluted with the liquid medium to reach a final bacterial count of approximately 1.5×10^6^ CFU/mL. The final concentrations of propolis ranged from 20 to 16 µg/mL in a series of two-fold dilutions. There were also control tubes with the liquid medium (without propolis) as negative controls and 80% ethanol and distilled water as positive controls. The MIC was defined as the lowest concentration that restricted the bacterial growth to an absorbance lower than 0.05 at 550 nm (invisible growth). 

For the determination of MBC, Sterile swabs were dipped into the tube that contained propolis concentrations higher than the MIC and inoculated onto the agar medium. The MBC was defined as the lowest concentration that allowed no visible growth on the agar.

*Statistical analysis*

The results were summarized as mean ± standard deviation and analyzed with SPSS (Version 16.0). The data were submitted to analysis of variance using ANOVA test. The significance chosen level was p < 0.05.

## Results and Discussion

As part of a continuing study on the prevention of dental diseases by natural drugs, we hypothesized that Iranian propolis, may be a valuable resource for the control of the oral biofilm and subsequent dental caries development.

In the present study, we first evaluated the antibacterial activity of the extracts using the agar-well diffusion method. Table 1 present the mean diameters of growth inhibition zones’ values (mm) obtained for each tested strain. The ethanolic extract of propolis (EEP) produced inhibitory zones against all the tested microorganisms. Among the strains tested with EEP, the most sensitive was *S. salivarius*, which showed the highest inhibition zones (20.00 ± 1.00 mm). 

**Table 1:** Susceptibility of oral bacteria against Iranian propolis

**Water extract (mm)**	**Ethanol extract (mm)**	**Microorganisms **
12 ± 1.00	16 ± 0.00	*S. mutans*
-	20 ± 1.00	*S. salivarius*
-	17 ± 0.00	*S. aureus*
11 ± 0.00	13 ± 1.00	*E. faecalis*
-	12 ± 1.00	*L. casei*

Inhibition zone values are given in mm (mean ± SD; n = 5). Negative control was inactive

The most resistant strain was *L. Casei *with growth inhibition zones of 12.00 ± 1.00 mm. Growth of the *S.mutans* and *Staph. aureus*was also significantly inhibited, confirming the previous results (11, 26). On the other hand, the water extract of Iranian propolis demonstrated only slight activity, in which 3 out of 5 strains presented no zone of growth inhibition. WEP showed slight activity only against *S. mutans *and* E. faecalis.* The results showed that, at a concentration of 20 mg/mL, EEP is more effective than WEP (p < 0.05). The negative controls (water and ethanol) did not show any inhibitory effects on the tested microorganisms.

The MICs and MBCs of the test substances are shown in Tables 2 and 3 for all strains. MIC was determined as the lowest concentration of the propolis extract, which inhibited the growth of the tested microorganisms (Table 2). The EEP showed a MIC and MBC in the range of 250-500 µg/mL. WEP, inhibited only the growth of*S. mutans *and *E. faecalis* (MIC = 500 µg/mL) and did not show any bactericidal activity at the concentrations used in this assay, with the exception of *S. mutans *(MBC of 20 mg/mL).

**Table 2:** Minimum inhibitory concentration of Iranian propolis extracts obtained for each tested strain

**Water extract MIC (µg/mL)**	**Ethanol extract MIC (µg/mL)**	**Microorganisms**
500	250	*S. mutans*
-	500	*S. salivarius*
-	250	*S. aureus*
500	250	*E. faecalis*
-	250	*L. casei*

The positive controls presented regular bacterial growth

**Table 3:** Minimum bactericidal concentration (MBC) of Iranian propolis extracts obtained for each tested strain

**Water extract MBC (mg/mL)**	**Ethanol extract MBC (µg/mL)**	**Microorganisms**
20	250	*S. mutans*
-	500	*S. salivarius*
-	500	*S. aureus*
-	500	*E. faecalis*
-	250	*L. casei*

The applied studies on the antimicrobial activity of propolis show different results (27). These conflictions in the antimicrobial activity of propolis could be due to the differences in its chemical components (27). It has also been reported that the samples collected from different geographic origin with different climates and vegetation show different antibacterial activities (28). Moreover, seasonal effect on Brazilian propolis antibacterial activity has been investigated with other researchers (29). 

The Iranian propolis extract used in this study showed proper antibacterial capacity, however, it is important to consider that *in-vitro* tests do not reproduce the real conditions of the oral cavity. In addition, determination of inhibition zones’ values depends on the technical details that are different in various laboratories. The size of the inhibition zones depends on the diffusibility of the test substance in the agar, which is under the influence of molecular weight, negative charge, composition of simples, and the thickness and pH of the agar culture medium (30). There are numerous questions yet to be answered concerning chemical compositions of Iranian propolis. Besides, the effects of the propolis extracts on the biofilm, need to be evaluated in an *in-vivo *model. 
